# Bringing Bike Share to a Low-Income Community: Lessons Learned Through Community Engagement, Minneapolis, Minnesota, 2011

**DOI:** 10.5888/pcd10.120274

**Published:** 2013-08-15

**Authors:** Sarah Kretman Stewart, David C. Johnson, William P. Smith

**Affiliations:** Author Affiliations: David C. Johnson, Hennepin County Human Services and Health Department, Minneapolis, Minnesota; William P. Smith, Biko Associates, Minneapolis, Minnesota.

## Abstract

**Background:**

High prevalence of physical inactivity contributes to adverse health outcomes. Active transportation (cycling or walking) is associated with better health outcomes, and bike-sharing programs can help communities increase use of active transportation.

**Community Context:**

The Minneapolis Health Department funded the Nice Ride Minnesota bike share system to expand to the Near North community in Minneapolis, Minnesota. Near North is a diverse, low-income area of the city where residents experience health disparities, including disparities in physical activity levels.

**Methods:**

The installation of new bike share kiosks in Near North resulted in an environmental change to support physical activity. Community engagement was conducted pre-intervention only and consisted of focus groups, community meetings, and interviews. Postintervention data on bike share trips and subscribers were collected to assess intervention effectiveness.

**Outcome:**

Focus group participants offered insights on facilitators and barriers to bike share and suggested system improvements. Community engagement efforts showed that Near North residents were positive about Nice Ride and wanted to use the system; however, the numbers of trips and subscriptions in Near North were low.

**Interpretation:**

Results show that the first season of the expansion was moderately successful in improving outreach efforts and adapting bike share to meet the needs of low-income populations. However, environmental change without adequate, ongoing community engagement may not be sufficient to result in behavior change.

## Background

The health benefits of physical activity are well-established, including prevention of weight gain and lowered risk of stroke, diabetes, and early death (1). However, only 49.7% of men and 46.7% of women in the United States (2) and 45.0% of Minnesotans (3) get sufficient leisure-time physical activity, and levels are lower among minorities and those with less education (2). At the same time, an estimated 35.7% of US adults (4) and 24.8% of Minnesotans (3) are obese. Disparities in obesity exist across racial/ethnic groups: an estimated 34.3% of non-Hispanic whites are obese, compared with 49.5% of non-Hispanic blacks and 37.9% of Hispanics (4). Among women, obesity is inversely associated with socioeconomic status, but no clear pattern exists among men (5).

Active transportation (ie, biking and walking to destinations) can increase physical activity. Active transportation is associated with better fitness, reduced risk for cardiovascular disease, and lower rates of obesity and diabetes (6,7). It may also result in cost savings: for example, if residents of Minnesota’s urban Twin Cities (Minneapolis and St. Paul) replaced half of short car trips with bike trips in warmer months, the estimated cost savings from avoided mortality and reduced health care costs are $146 million per year (8). Despite these benefits, only 0.6% of Americans commute by bicycle, and no evidence of differences is seen by race, ethnicity, or income (9).

Public health advocates have embraced bike share as a way to increase active transportation. Bike share users pay a small fee to check out bikes from kiosks for short periods of time. In Barcelona, researchers found that the health benefits gained through use of bike share outweigh the risks (10). Other researchers found that exposure to bike share was associated with increased cycling in Montreal (11). Many cities have launched bike share, including the Twin Cities, where Nice Ride Minnesota was launched in 2010.

## Community Context

**Figure 1:**
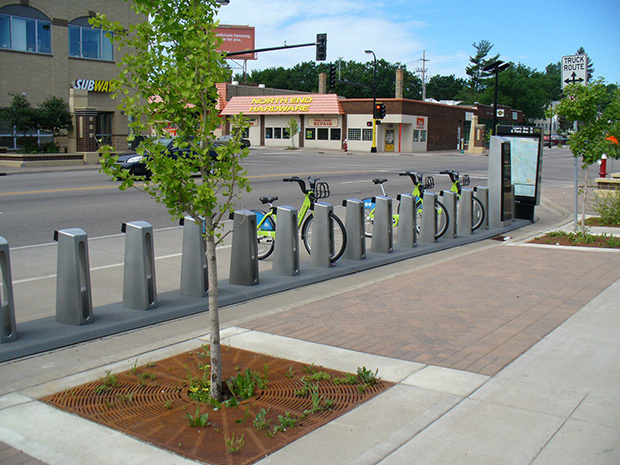
Bike share kiosks in the community of Near North, Minneapolis, Minnesota.

To increase physical activity opportunities, the Minneapolis Health Department (MHD), through its Communities Putting Prevention to Work (CPPW) grant, funded an expansion of Nice Ride into Near North, Minneapolis (Figure 1). To use Nice Ride, subscribers pay a small fee join the network for 24 hours, 1 month, or 1 year, and can use bikes to make short trips of 30 minutes or less for no additional fee.

Near North is a diverse community of 31,192 with a median household income of $32,413 and 40% of residents living below the federal poverty level, compared with a citywide median household income of $45,625 and 22% of residents living below the federal poverty level (12). Near North residents are African American (52.4%), white (17.5%), Asian (14.9%), Hispanic (8.5%), American Indian (1.3%), and 2 or more races (5.2%) (12). In North Minneapolis (the quadrant of the city that includes Near North), 30% of residents are obese, 22% report having hypertension, and 8% report having diabetes (compared with 19%, 15%, and 5% of the city as a whole, respectively). Only 28% meet the Healthy People 2010 Guidelines for Physical Activity (compared with 38% of the city as a whole) (13).

Minneapolis is a bike-friendly city with more than 167 miles of bikeways (14). Metro Transit offers public transportation that includes a network of regular-route buses, light rail, and commuter rail. According to 2006–2010 American Community Survey estimates, 3.7% of residents commute to work by bicycle (13); however, bicyclists are not evenly distributed throughout the city. Annual bicycle count data collected by the city and a local nonprofit show that the lowest bicycle volumes are in Near North, where only 9 bicyclists were counted during a 2-hour period at a Near North location, compared with 2,627 cyclists in the highest count location, near a large university (15). Bicycling is uncommon even though 31.2% of Near North households do not have access to a vehicle (12). Bicycle infrastructure (eg, bike lanes and signs) has been added recently to Near North, which may partially explain the lower bike counts. Near North is also cut off from the rest of the city by 2 interstates and the Mississippi River.

In response to the health disparities experienced by Near North residents, MHD focused much of its CPPW grant on increasing opportunities for physical activity in Near North, including expanding Nice Ride. The expansion was also a response to residents’ desire for the program. In the initial launch of Nice Ride in 2010, no kiosks were placed in Near North, which has low housing density and fewer popular destinations. Instead, as in other bike share programs, the initial network was focused in areas with high concentrations of people and destinations. Some Near North residents and elected officials expressed frustration that no kiosks were initially placed in their community (16), and Nice Ride program leaders responded by placing 3 kiosks in Near North in 2010.

In 2011, Nice Ride used funds from CPPW and a local nonprofit to expand the Near North network to 11 kiosks, to increase physical activity among residents. At the end of 2011, the entire Nice Ride system had 116 kiosks, and users took more than 217,000 trips (14). Before the 2011 expansion, a Near North–based planning firm led a community engagement process to gather input to inform the expansion. To assess the effectiveness of the bike-share expansion, MHD tracked new subscriptions among Near North residents and the number of rides to and from Near North kiosks during the 2011 season (April–November). Other researchers have found lower uptake of bike share among populations with low levels of income (17) and education (18); however, to our knowledge, no studies have reported qualitative data about perceptions of bike share in low-income populations.

## Methods

### Community engagement efforts

MDH provided approximately $27,000 to the planning firm to conduct pre-intervention community engagement to increase existing support and gather input from community residents. The planning firm worked with MHD and Nice Ride to design the community engagement process, facilitate the community meetings, conduct the focus groups, and conduct interviews with elected officials. Community engagement activities after the expansion were limited on the assumption that increased access to Nice Ride kiosks (an environmental change) would lead to increased cycling for some residents. Therefore, focused community engagement happened only before the intervention and concentrated on ensuring that the environmental change met community needs and expectations.

Community engagement lasted from October 2010 through January 2011 beginning with a community meeting to introduce the expansion to the community and gather input (October), followed throughout the fall by focus groups and interviews with elected officials representing Near North. Findings were presented in January at a final community meeting. A technical advisory committee (TAC) with representatives from Nice Ride, MHD, the planning firm, and a social service organization oversaw the process.

Community meetings were promoted through neighborhood organizations and advertisements in community newspapers. To ensure that focus groups were diversified, recruitment was conducted in partnership with a business association, an African American bicycle advocacy group, and social service organizations with connections to community members. Recruitment partners used personal conversations and fliers to reach community members. Postcards describing the project and asking for input were also distributed to businesses along a commercial street in Near North. The advertisements, fliers, and postcards were developed by the planning firm with feedback from the TAC.

Six audience-specific focus groups were conducted with the following audiences: local business owners; employees of local businesses; representatives from nonprofit institutions; clients of social service organizations; Near North bicycle advocates; and residents of low-income housing and students at a vocational training institution. Each focus group had 12 participants, all of whom lived or worked in Near North. The focus group protocol was based on the 4-step ORID (objective, reflective, interpretive, and decisional) approach (19) and included an overview of Nice Ride, followed by asking about general impressions of the system, barriers to using the system, ideas for improving and marketing the system, and willingness to use the system. Participants received a small gift card, a meal, and a T-shirt as incentives for participation. The planning firm staffers took detailed notes, analyzed them, and summarized them into themes. Focus groups were not recorded or transcribed, and demographic information was not collected. Community members were not directly involved in data analysis or reporting.

The planning firm analyzed the summarized community engagement results into a presentation shared at the second community meeting and a final report made available online, both of which were shared with MDH public health practitioners. Public health practitioners also attended the community meetings.

### Bike share subscriber and kiosk usage data

From the system launch in April 2011 until the season end in November 2011, we gathered 2 types of data to assess residents’ use of the system: kiosks usage and subscriber data. Nice Ride data were collected by a third-party system administrator and then shared with Nice Ride, whose staff members stripped data files of personally identifying information and provided them to MHD electronically. An MHD staff member analyzed data by using SPSS 19.0.0 (IBM Corp, Armonk, New York).

Nice Ride has 3 types of subscribers: annual and 30-day subscribers, who purchase yearlong subscriptions online, and casual subscribers, who purchase 24-hour subscriptions at kiosks. Data collected from annual subscribers when they purchase their subscriptions are credit card information, sex, birth date, address, and subscription price; a zip code and a unique identification number associated with a credit card are collected from casual subscribers.

Nice Ride collects the following data on trips (anytime a bike is checked out of a kiosk): starting kiosk, ending kiosk, duration, cost, customer zip code, and a unique identification number associated with the customer credit card. Data were used to determine the total number of trips in the system, number of trips to or from Near North kiosks, and the number of trips by subscribers with Near North zip codes. Trips that began and ended at CPPW kiosks were coded as internal to internal, trips that originated at a non-CPPW kiosks and ended at a CPPW kiosk were coded as external to internal, trips that originated at a CPPW kiosk and ended at a non-CPPW kiosk were coded as internal to external, and other trips that did not involve CPPW kiosks were coded as external. Analysis included descriptive statistics to examine the number of trips involving CPPW kiosks and cross-tabulations to compare trips involving CPPW kiosks with those of the external network. The average and total durations of CPPW kiosk trips were calculated and used to estimate calories burned (based on an average of 290 calories burned per hour of moderately paced cycling) (20).

## Outcome

### Pre-intervention community engagement results

Community meeting attendees, elected officials, and focus group participants viewed Nice Ride positively. Many suggestions for kiosk locations overlapped across focus groups and were similar to suggestions offered by community meeting participants and elected officials. In the first community meeting and interviews, residents and elected officials described potential barriers to accessing bike share, including confusion about how the bike share system worked, lack of access to computers and credit cards, and community perceptions of unsafe bicycling conditions. Community meeting participants also identified personal safety concerns and the 30-minute time limit as potential barriers. The second community meeting was dedicated to presenting the input back to the community.

Most focus group participants said they would buy a subscription. Participants offered comments on the benefits of bike share, barriers to using it, and suggestions for conducting outreach and improving the system (Box 1).

Box 1 **Summary of Themes From Near North Focus Group Participants About the Nice Ride Bike Share System, Minneapolis, Minnesota, 2011**

Benefits of Nice Ride

- Nice Ride is a community asset that increases community participation and connections.
- Nice Ride is a way to get physical activity and recreation, more than a mode of transportation.
- Nice Ride is convenient for errands and other short trips.
- The price of an annual subscription ($60) is reasonable and competitive with transit.
- Nice Ride bikes are not likely to get stolen because they lock securely at kiosks and are an identifiable (lime-green) color.

Barriers to using Nice Ride

- The credit card requirement excludes some people from participating, and people who have credit or debit cards might not want to use them to purchase a subscription.
- The 30-minute time limit is not long enough.
- Lack of computer access could prevent people from purchasing subscriptions.
- The $60 annual subscription cost was too high to some, others felt that the fee could not be made in one payment, and the $5 daily subscription fee was viewed as too high and uncompetitive with the bus.
- To some, the system was confusing to sign up for and use.
- Streets were not perceived to be bike-friendly (even streets with bike lanes).
- Bikes are not made to haul much cargo or bring children along.
- Clients of social services organizations associated bicycle use with professional business people, whereas students and residents of low-income housing associated bicycle use with lack of success.

Ideas for program improvements

- Partner with community organizations and agencies to increase awareness of Nice Ride and its benefits and to get discounted subscriptions to low-income individuals.
- Conduct targeted outreach: use images of Near North residents and destinations; in advertisements; hold community events.
- Accommodate recreational use by increasing the trip-time limit (at least for some bikes).
- Make subscriptions more accessible and convenient to purchase by selling them at local stores.
- Connect bike share systems with transit systems so they can be used as an integrated system.

Nice Ride installed kiosks at or near most of the locations suggested by the community. In response to community input, Nice Ride hired a staff person who spent part of her time working to develop partnerships with community organizations serving low-income populations as a way to distribute discounted $20 annual subscriptions. (At the time, full-price annual subscriptions cost $60, and daily subscriptions cost $5. Nice Ride also offered sale-priced annual subscriptions for $40 to anyone who joined the network in April and May 2011.) Nice Ride staff contacted potential partners through existing connections, cold calls, and e-mails and offered to talk about the program and lead events and bike rides with clients. This process resulted in a few partnerships and events but almost no subscriptions. Likely barriers to success include the lack of follow-up with participants after events and the limited time that Nice Ride and community organization staff had to dedicate to this initiative. A challenge to community outreach was that MHD funding covered only the preseason engagement work and the installation of the kiosks. Funds did not support ongoing community engagement throughout the season because the focus of the project was getting the environmental change (bike share) in place.

An unexpected challenge encountered was a tornado that passed through Near North on May 22, 2011. None of the Nice Ride kiosks were damaged, but many houses and apartments in the area were affected, as were many residents. Residents and community organizations were focused on rebuilding, and Nice Ride was not a priority.

### Postintervention trip and subscriber results

In 2011, the entire network had more than 3,000 long-term (annual and 30-day) and 37,000 casual subscribers, including 124 annual and 208 casual subscribers in North Minneapolis, which includes Near North. Most annual subscriptions to Northside residents were sale subscriptions (88%) costing $40 per year. Annual subscribers accounted for 88% of trips taken by North Minneapolis residents compared with 68% of trips in the system overall. Near North subscribers took a total of 2,741 trips throughout the whole network during the 2011 season, and North Minneapolis annual subscribers took an average of 8.2 trips each. The average number of trips cannot be calculated for casual subscribers because casual subscriber data is tied to a credit card, not to an individual.

**Figure 2:**
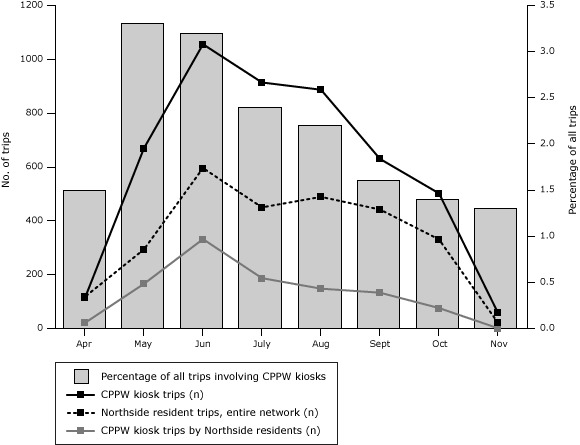
Nice Ride bike share trip and subscriber data.

A total of 217,530 bike trips were made in the entire Nice Ride bike system in 2011; 4,831 (2.2%) of all trips were to or from 1 of the 8 kiosks funded by this project. During the season, 22% of the CPPW kiosk trips (or 1,064 trips) were taken by North Minneapolis residents (Figure 2).

**Table:** 

Characteristic	Apr	May	June	July	Aug
**CPPW kiosk trip**	114	670	1,056	914	887
**Northside resident trips (whole network)**	118	294	596	450	489
**CPPW kiosk trip, Northside resident **	21	166	332	187	148
**Percentage of trips involving CPPW kiosks**	1.50%	3.30%	3.20%	2.40	2.20%

Abbreviation: CPPW, Communities Putting Prevention to Work.

North Minneapolis subscribers were more likely to start or end trips outside Near North than within the area. Most CPPW kiosk trips (84.8%) started or ended outside of the CPPW network. All CPPW kiosks were located in Near North, and the project kiosk with the highest usage was near a commuter parking lot on the edge of downtown.

The average duration of CPPW kiosk trips was 22.2 minutes, and the total duration was 1,789 hours. Based on estimates of caloric expenditure from biking at a moderate pace, and assuming that bike share users cycled the entire time they had checked out bikes, we calculate that CPPW-kiosk users burned about 107 calories per trip, on average, or 518,000 calories in total (19). However, because annual subscribers took an average of 8.2 trips, their contribution to their overall energy expenditure may have been minimal.

## Interpretation

The 2011 season in Near North was moderately successful but revealed that simply installing kiosks in a low-income area is not sufficient to increase residents’ use of bike share. The average trip duration of 22.2 minutes provides riders with more than two-thirds of daily recommended physical activity levels, which demonstrates the potential of bike share to contribute to physical activity of regular users.

Use of the CPPW-funded Nice Ride kiosks peaked in the early summer, and the percentage of total trips taken at CPPW kiosks fell during the summer because the overall system expanded and CPPW kiosks represented a declining percentage of the entire system. Near North kiosk usage and subscriptions were much lower than in other areas, possibly because Near North is more isolated, underdeveloped, and separated from active and densely developed areas by freeways, and Near North’s main commercial corridor is a street without clustered commercial destinations. Nice Ride trips have been higher in areas of the city with desirable destinations, such as in downtown and near a large university. Financial, awareness, knowledge, or self-efficacy barriers to using Nice Ride faced by Near North residents may have also contributed to low numbers.

These results show that in low-income areas without many desirable destinations, communities should consider focusing primary marketing and promotions efforts on residents who work, go to school, or shop outside the area, where users may be more likely to take trips. Designers of marketing and promotions campaigns for bike share should carefully define and target intended markets, define bike share as a transportation option with competitive advantages and coincident health benefits and as a recreational resource with coincident transportation benefits, and create promotional advertisements that target communities that racial/ethnic minorities can relate to.

Results of the focus groups also suggest that accommodations should be made in the system to meet the needs of low-income individuals (Box 2). Nice Ride is considering 2 strategies for low-income populations: 1) extending trip time limits from 30 minutes to 45 minutes and 2) installing more stations at destinations for recreational riding (eg, lakes and parks). Nice Ride is also experimenting with the use of prepaid debit cards for low-income individuals who sign up using free subscriptions, but these subscriptions were not offered at the time of the community engagement described in this article.

Box 2 **Potential Strategies for Accommodating Low-Income Users of Bike Share**

Lower-cost strategies

- Create opportunities for people to try riding bikes, such as offering tours.
- Offer long-term subscriptions for purchase at convenient locations (not just online).
- Implement long-term community engagement efforts in partnership with community organizations.
- Offer low-cost subscriptions to low-income individuals, potentially through partnerships with community organizations.
- Target promotional materials to low-income populations (eg, by using images that reflect their demographics, by featuring destinations in those communities).

Higher-cost strategies

- Develop a system to accept alternative forms of payment besides debit or credit cards.
- Integrate bike share with public transit: allow transit passes and bike share subscriptions to be purchased together; create one card or key fob to access both systems.
- Offer bikes that can haul cargo (eg, groceries).
- Offer bikes that can safely carry young children with an adult rider.

The results of this project also show that an environmental change in a community (in this case, bike share) may not be sufficient to lead to behavior change within a low-income population. The community should be engaged in the project on an ongoing basis before and after the change is made. This finding serves as a reminder for communities (including Minneapolis) that focus on environmental change work.

This analysis has several limitations. Only 1 year of data was available, preventing year-to-year comparisons. More time may also be needed for low-income residents to become bike share users. Focus group results may not reflect the opinions of the general population. Furthermore, because no additional data on bike share users’ physical activity were available, we could not determine whether bike share increased physical activity levels for users or replaced other forms of physical activity in which bike share users had already been engaging. Future practice-based evaluations may show what strategies are most effective in encouraging low-income populations to use bike share.
